# Supplementary material for: Versatile seamless DNA vector production in E. coli using enhanced phage lambda integrase
Source: PLoS One. 2022 Sep 23;17(9):e0270173. doi: 10.1371/journal.pone.0270173 (PMC9506625; doi:10.1371/journal.pone.0270173)

Related to main Figure 3B
Mini seamless vector production
Lane numbers refer to main Figure Legend
X: unrelated (or empty lanes); L: marker lane

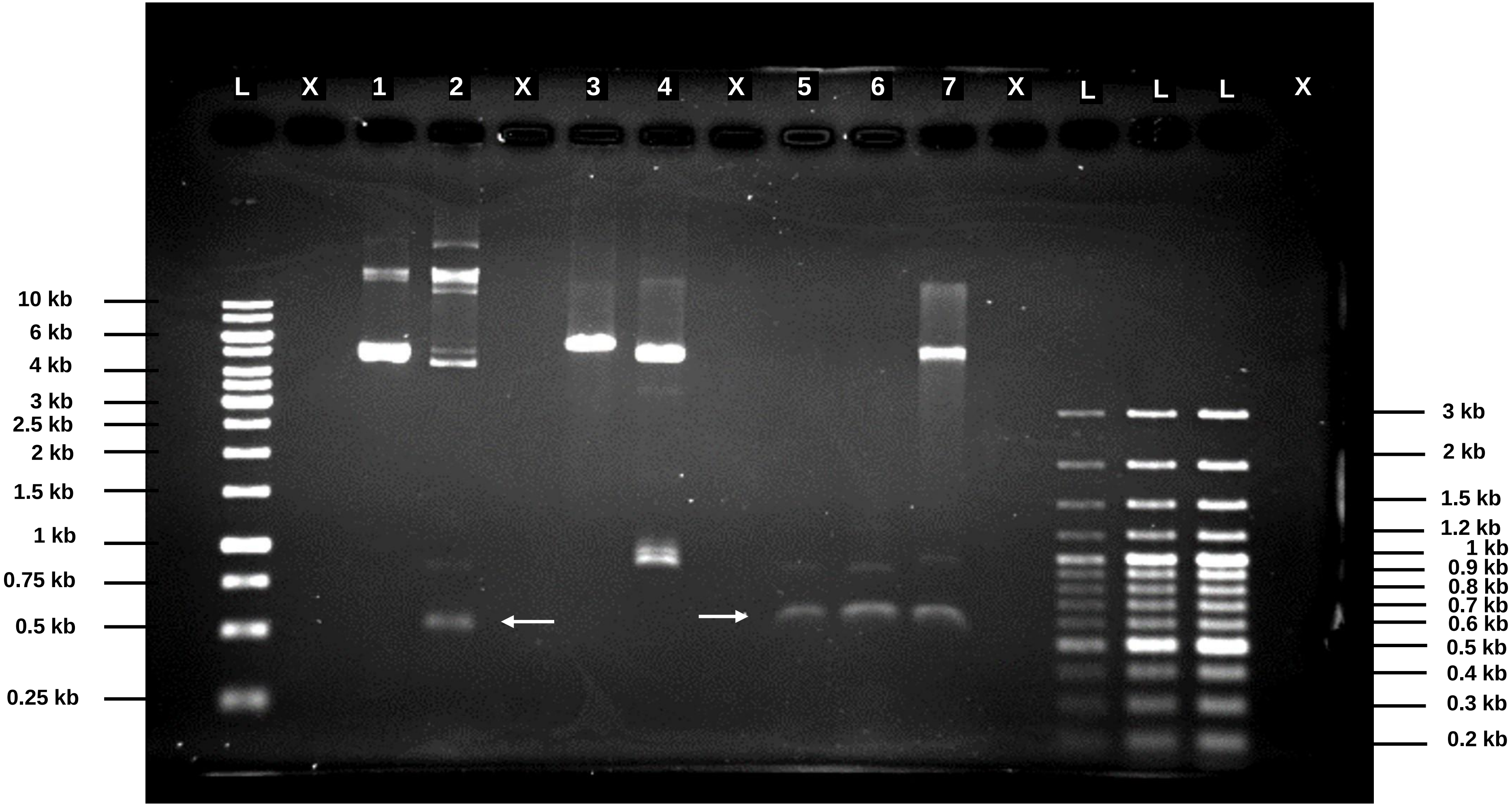

Related to main Figure 4B
Large seamless vector production
Lane numbers refer to main Figure Legend
X: unrelated (or empty lanes); L: marker lane

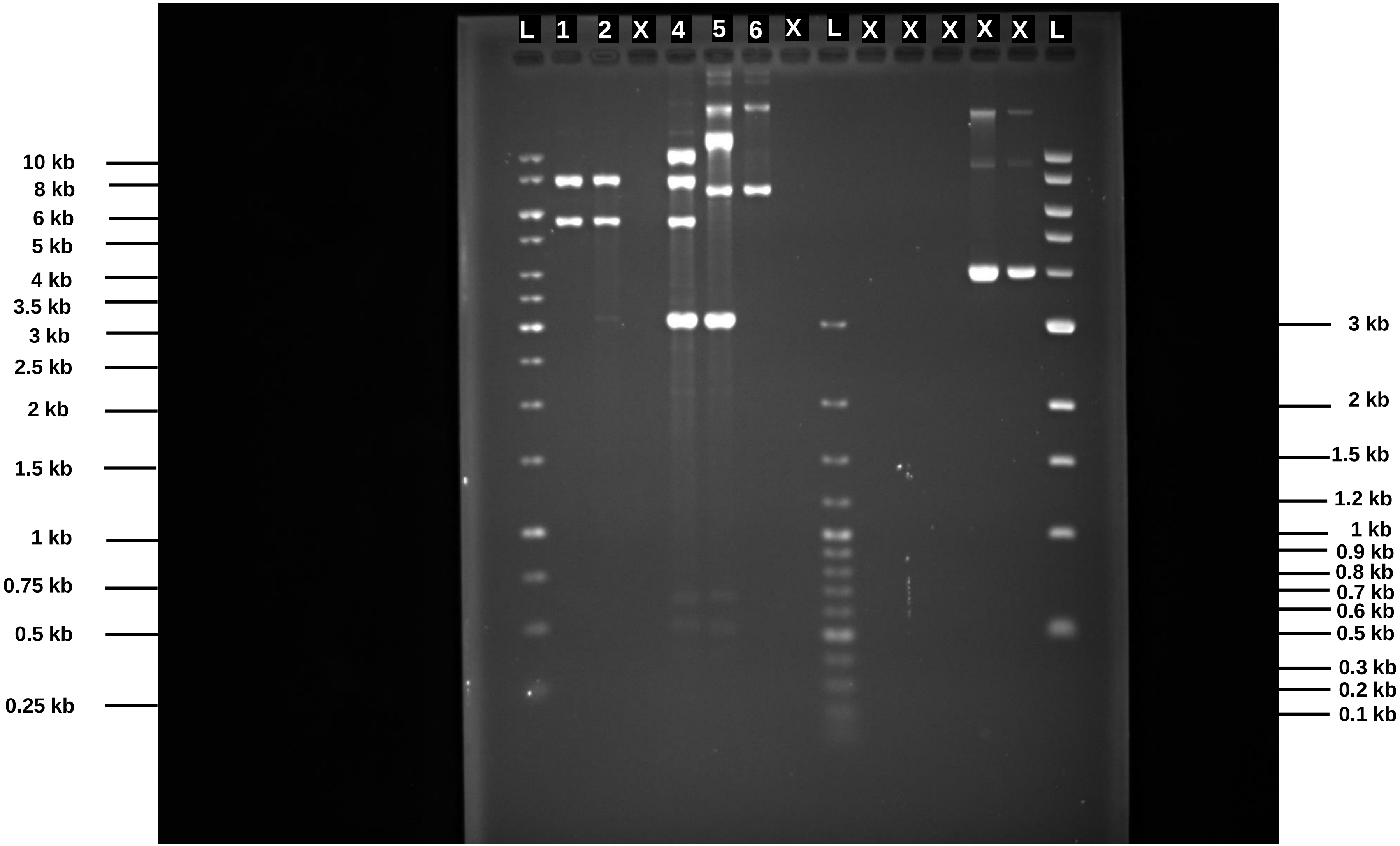

Related to Figure S2 A,B
Left and right junction PCR

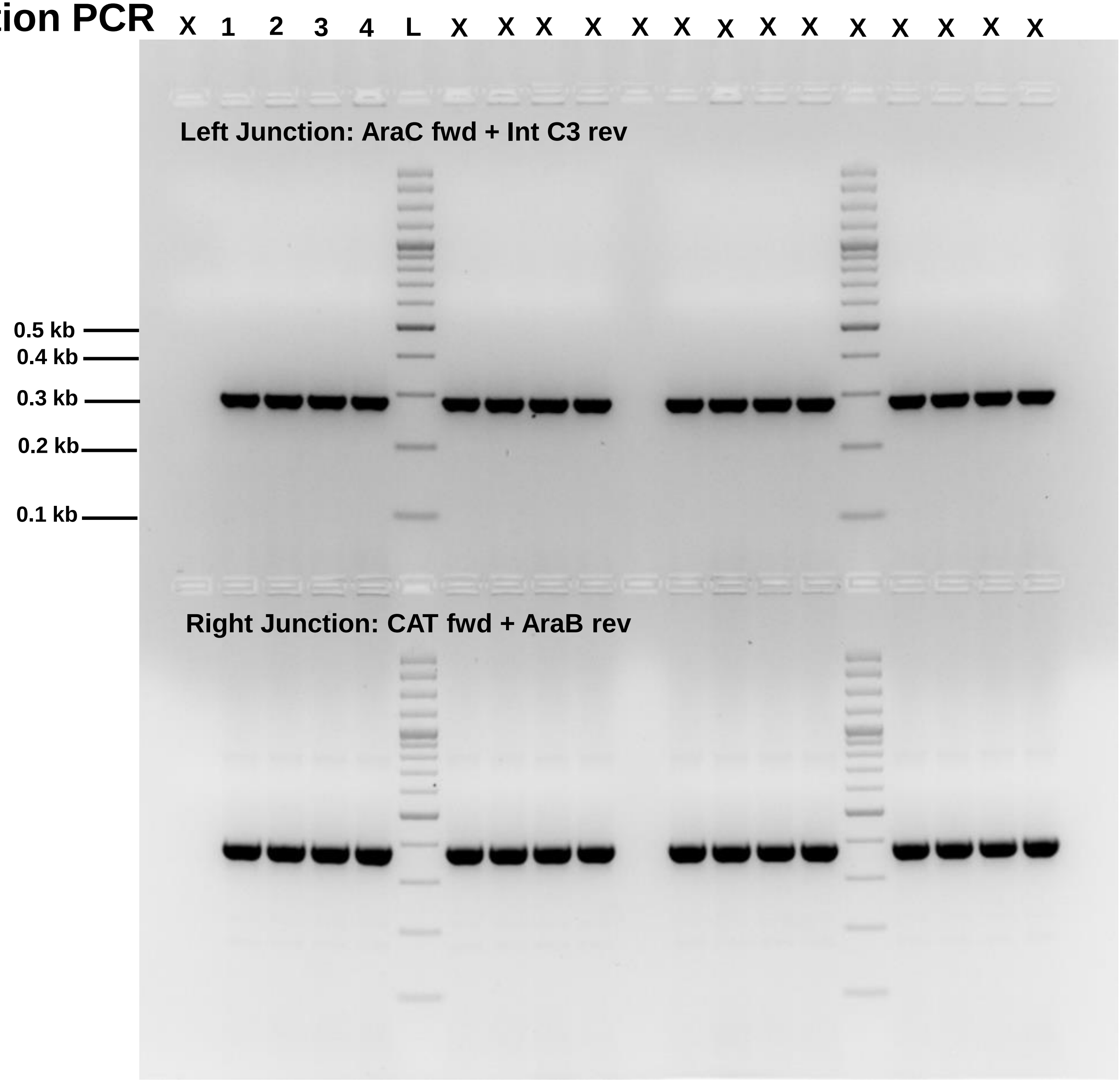

Related to Figure S2C
Genomic PCR

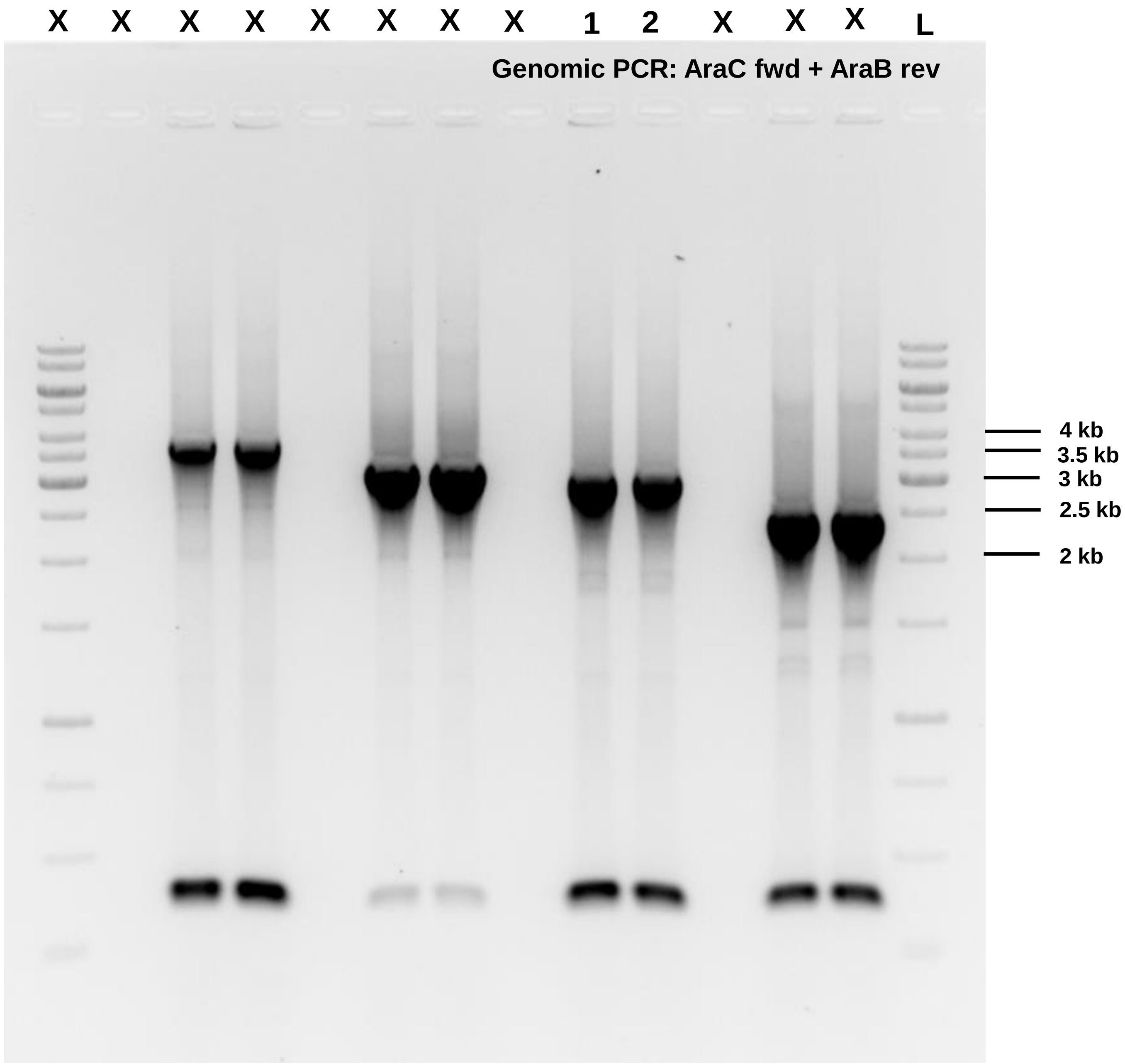

Supplement: S1 Raw image — (PDF) [file pone.0270173.s001.pdf]
