# Supplementary material for: Versatile seamless DNA vector production in E. coli using enhanced phage lambda integrase
Source: PLoS One. 2022 Sep 23;17(9):e0270173. doi: 10.1371/journal.pone.0270173 (PMC9506625; doi:10.1371/journal.pone.0270173)
Supplement: S1 Fig — See text for details. (PPTX) [file pone.0270173.s002.pptx]

## Slide 1
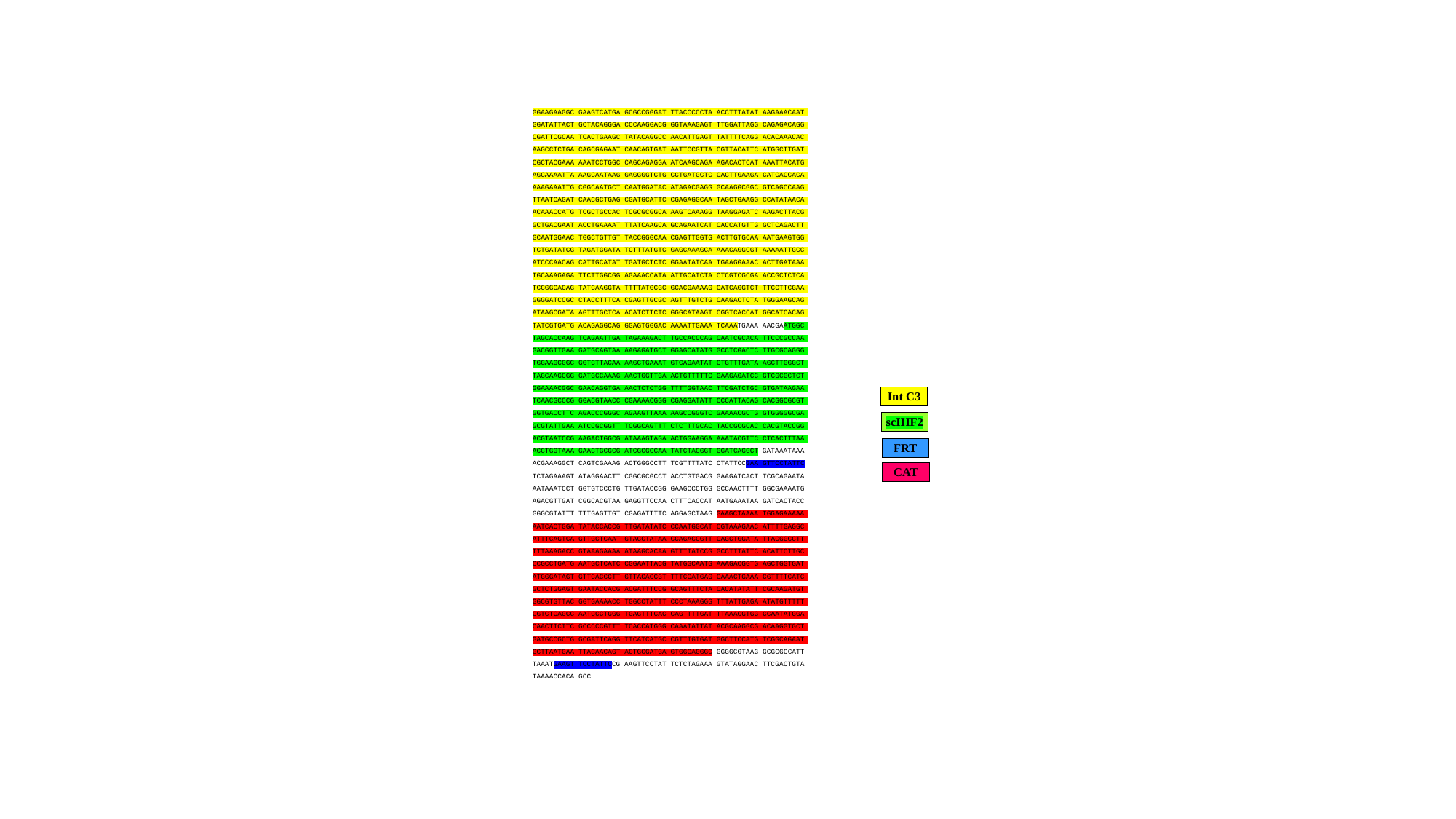

GGAAGAAGGC GAAGTCATGA GCGCCGGGAT TTACCCCCTA ACCTTTATAT AAGAAACAAT
GGATATTACT GCTACAGGGA CCCAAGGACG GGTAAAGAGT TTGGATTAGG CAGAGACAGG
CGATTCGCAA TCACTGAAGC TATACAGGCC AACATTGAGT TATTTTCAGG ACACAAACAC
AAGCCTCTGA CAGCGAGAAT CAACAGTGAT AATTCCGTTA CGTTACATTC ATGGCTTGAT
CGCTACGAAA AAATCCTGGC CAGCAGAGGA ATCAAGCAGA AGACACTCAT AAATTACATG
AGCAAAATTA AAGCAATAAG GAGGGGTCTG CCTGATGCTC CACTTGAAGA CATCACCACA
AAAGAAATTG CGGCAATGCT CAATGGATAC ATAGACGAGG GCAAGGCGGC GTCAGCCAAG
TTAATCAGAT CAACGCTGAG CGATGCATTC CGAGAGGCAA TAGCTGAAGG CCATATAACA
ACAAACCATG TCGCTGCCAC TCGCGCGGCA AAGTCAAAGG TAAGGAGATC AAGACTTACG
GCTGACGAAT ACCTGAAAAT TTATCAAGCA GCAGAATCAT CACCATGTTG GCTCAGACTT
GCAATGGAAC TGGCTGTTGT TACCGGGCAA CGAGTTGGTG ACTTGTGCAA AATGAAGTGG
TCTGATATCG TAGATGGATA TCTTTATGTC GAGCAAAGCA AAACAGGCGT AAAAATTGCC
ATCCCAACAG CATTGCATAT TGATGCTCTC GGAATATCAA TGAAGGAAAC ACTTGATAAA
TGCAAAGAGA TTCTTGGCGG AGAAACCATA ATTGCATCTA CTCGTCGCGA ACCGCTCTCA
TCCGGCACAG TATCAAGGTA TTTTATGCGC GCACGAAAAG CATCAGGTCT TTCCTTCGAA
GGGGATCCGC CTACCTTTCA CGAGTTGCGC AGTTTGTCTG CAAGACTCTA TGGGAAGCAG
ATAAGCGATA AGTTTGCTCA ACATCTTCTC GGGCATAAGT CGGTCACCAT GGCATCACAG
TATCGTGATG ACAGAGGCAG GGAGTGGGAC AAAATTGAAA TCAAATGAAA AACGAATGGC
TAGCACCAAG TCAGAATTGA TAGAAAGACT TGCCACCCAG CAATCGCACA TTCCCGCCAA
GACGGTTGAA GATGCAGTAA AAGAGATGCT GGAGCATATG GCCTCGACTC TTGCGCAGGG
TGGAAGCGGC GGTCTTACAA AAGCTGAAAT GTCAGAATAT CTGTTTGATA AGCTTGGGCT
TAGCAAGCGG GATGCCAAAG AACTGGTTGA ACTGTTTTTC GAAGAGATCC GTCGCGCTCT
GGAAAACGGC GAACAGGTGA AACTCTCTGG TTTTGGTAAC TTCGATCTGC GTGATAAGAA
TCAACGCCCG GGACGTAACC CGAAAACGGG CGAGGATATT CCCATTACAG CACGGCGCGT
GGTGACCTTC AGACCCGGGC AGAAGTTAAA AAGCCGGGTC GAAAACGCTG GTGGGGGCGA
GCGTATTGAA ATCCGCGGTT TCGGCAGTTT CTCTTTGCAC TACCGCGCAC CACGTACCGG
ACGTAATCCG AAGACTGGCG ATAAAGTAGA ACTGGAAGGA AAATACGTTC CTCACTTTAA
ACCTGGTAAA GAACTGCGCG ATCGCGCCAA TATCTACGGT GGATCAGGCT GATAAATAAA
ACGAAAGGCT CAGTCGAAAG ACTGGGCCTT TCGTTTTATC CTATTCCGAA GTTCCTATTC
TCTAGAAAGT ATAGGAACTT CGGCGCGCCT ACCTGTGACG GAAGATCACT TCGCAGAATA
AATAAATCCT GGTGTCCCTG TTGATACCGG GAAGCCCTGG GCCAACTTTT GGCGAAAATG
AGACGTTGAT CGGCACGTAA GAGGTTCCAA CTTTCACCAT AATGAAATAA GATCACTACC
GGGCGTATTT TTTGAGTTGT CGAGATTTTC AGGAGCTAAG GAAGCTAAAA TGGAGAAAAA
AATCACTGGA TATACCACCG TTGATATATC CCAATGGCAT CGTAAAGAAC ATTTTGAGGC
ATTTCAGTCA GTTGCTCAAT GTACCTATAA CCAGACCGTT CAGCTGGATA TTACGGCCTT
TTTAAAGACC GTAAAGAAAA ATAAGCACAA GTTTTATCCG GCCTTTATTC ACATTCTTGC
CCGCCTGATG AATGCTCATC CGGAATTACG TATGGCAATG AAAGACGGTG AGCTGGTGAT
ATGGGATAGT GTTCACCCTT GTTACACCGT TTTCCATGAG CAAACTGAAA CGTTTTCATC
GCTCTGGAGT GAATACCACG ACGATTTCCG GCAGTTTCTA CACATATATT CGCAAGATGT
GGCGTGTTAC GGTGAAAACC TGGCCTATTT CCCTAAAGGG TTTATTGAGA ATATGTTTTT
CGTCTCAGCC AATCCCTGGG TGAGTTTCAC CAGTTTTGAT TTAAACGTGG CCAATATGGA
CAACTTCTTC GCCCCCGTTT TCACCATGGG CAAATATTAT ACGCAAGGCG ACAAGGTGCT
GATGCCGCTG GCGATTCAGG TTCATCATGC CGTTTGTGAT GGCTTCCATG TCGGCAGAAT
GCTTAATGAA TTACAACAGT ACTGCGATGA GTGGCAGGGC GGGGCGTAAG GCGCGCCATT
TAAATGAAGT TCCTATTCCG AAGTTCCTAT TCTCTAGAAA GTATAGGAAC TTCGACTGTA
TAAAACCACA GCC
Int C3
scIHF2
FRT
CAT
