# Supplementary figures and images for: Versatile seamless DNA vector production in E. coli using enhanced phage lambda integrase
Source: PLoS One. 2022 Sep 23;17(9):e0270173. doi: 10.1371/journal.pone.0270173 (PMC9506625; doi:10.1371/journal.pone.0270173)

## Slide 1
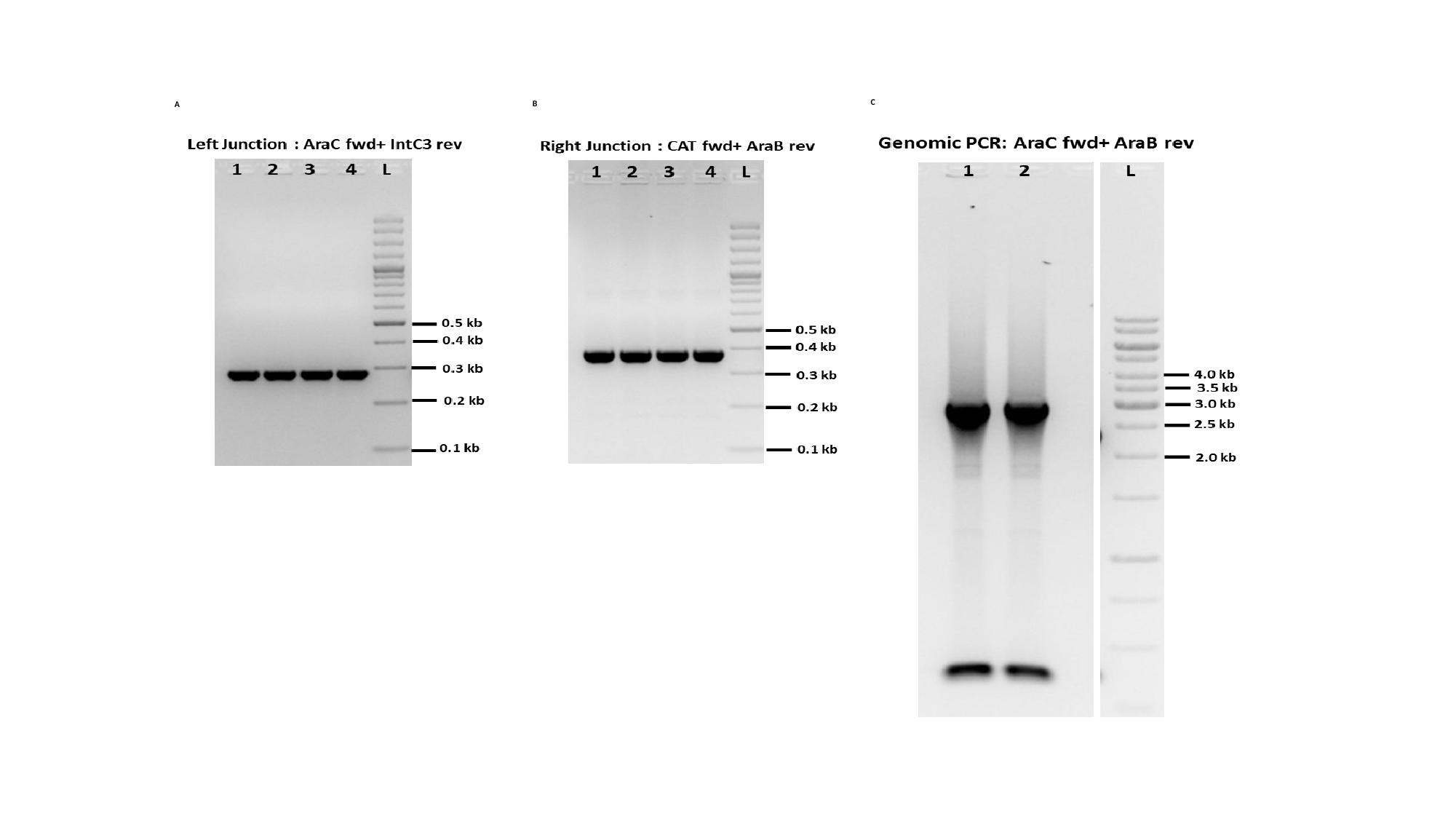

C
B
A

Supplement: S2 Fig — (A) Colony PCR was performed with primers ARAC_FWD and INTC3_REV for left junction. PCR amplified products of the expected size 291 bp were detected in all colonies. (B) Colony PCR was performed with primers CAT_FWD and ARAB_REV for right junction. PCR amplified products of the expected size 397 bp were detected in all colonies. (C) Genomic PCR was performed with primers ARAC_FWD and ARAB_REV for full insertion amplification. PCR amplified products of the expected size 2.8 kb were detected in two colonies. L: 100 bp ladder; Lanes 1–4: MG1655/pKD46 colonies electroporated with ISC PCR-amplified construct. (PPTX) [file pone.0270173.s003.pptx]

## Slide 1
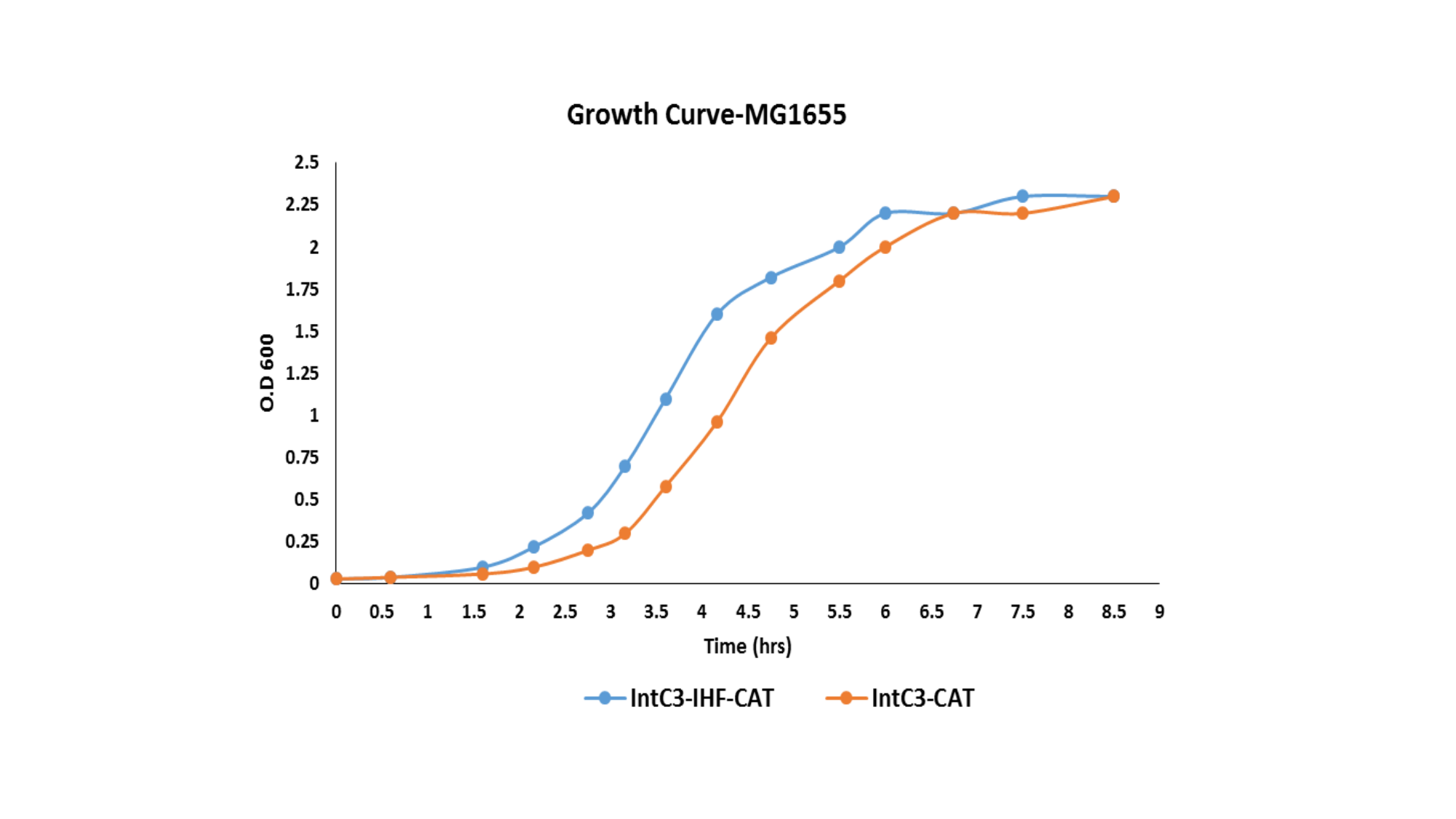

Supplement: S3 Fig — Cells of both strains in stationary phase were inoculated in fresh media and OD600 measured at different time points as indicated. Both strains show very similar exponential growth rates and cell densities in late stationary phase. (PPTX) [file pone.0270173.s004.pptx]
