# Supplementary material for: Versatile seamless DNA vector production in E. coli using enhanced phage lambda integrase
Source: PLoS One. 2022 Sep 23;17(9):e0270173. doi: 10.1371/journal.pone.0270173 (PMC9506625; doi:10.1371/journal.pone.0270173)
Supplement: Attachment — Submitted filename: Response to Reviewers.docx [file pone.0270173.s005.docx]

Date: 03-09-2022

**Response to Reviewers**

We would like to thank the reviewers for his/her thoughtful comments and efforts towards improving the manuscripts.

In the following sections, we address each point raised by the reviewers. All page numbers mentioned refer to the amended manuscript.

**Reviewer #1: Comments:**

1. Line 53-54: ‘However, employing these systems reproducibly and at larger scales remains technically challenging.’ Could the authors give further explanations on the challenges? I could not go there basing on the information provided by the authors.

We have included the explanation in the amended manuscript in line 54-56.

2. Line 61: Could the author give enough features on the ‘flexibility in att sites’? It is the main ‘novelty’ of this method and would be quite useful for other people to design their DNA sequences basing on this method.

“*att*” sites are flexible because not only *attP* but also *attL* can also recombine with *attH* for site specific recombination in human genome (Chandra *et al.*, 2016; Makhija *et al.*, 2018). Thus, the parental plasmids can be designed with different combinations of *att* sites for seamless vector production

3. Line 70: I do not know the relationship between the tightly regulated expression of ara operon and minimal prior genetic changes. Could the authors give some proofs or references?

Since MG1655 is cured of temperate lambda bacteriophage, hence there will be no leaky expression of integrase from the integrated lambda prophage (mentioned in line 73-75). In addition, as stated, we reasoned those minimal genetic changes will contribute to the functionality of the ara operon. We amended this in line 71 to “hypothesized”.

4. Line 73: The genome of MG1655 has some gene mutations except λ- and F-. ‘K-12 F– λ– ilvG– rfb-50 rph-1’ This genotype feature is derived from website of openwetware.

The mutations listed in the genotype are present in most K-12 strains and were probably acquired early in the history of the laboratory strain. A frameshift at the end of *rph* results in decreased *pyrE* expression and a mild pyrimidine starvation, such that the strain grows 10 to 15% more slowly in pyrimidine-free medium than in medium containing uracil (Jensen, 1993). The *ilvG*- mutation is a frameshift that knocks out acetohydroxy acid synthase II (Lawther *et al.*, 1982). The *rfb*-50 mutation is an IS5 insertion that results in the absence of O-antigen synthesis (Liu and Reeves, 1994).

5. In the result part, the authors also used the protein scIHF2. I suggested the authors also described its background and purpose in the introduction part.

We have followed this suggestion and incorporated the background of protein scIHF2 in the amended manuscript in line 78-80.

6. The sequences termed ISC was not long enough in Fig. S1. I also suggested the authors marked the names of each gene or element with underlines.

We have followed this suggestion and incorporated this change in Figure S1.

7. I suggested the authors could give a detailed protocol in the method section besides the development of MG1655-ISC.

The detailed protocol for seamless vector preparation has been already mentioned in line 154-169.

8. After the authors got the minicircles in the enzyme reaction buffer, dose it necessary to be purified again or direct to use with these complex enzymes and reagents? If purification is necessary, does the authors calculate the yield before purification or after purification? In addition, I do not know how to calculate minicircles yield in the process. Could the authors give detailed calculation process? (Line 211) In further, if purification is necessary, I also suggested the author gave a gel picture in Fig. 3 and Fig. 4 to show if it is possible the get ‘clean’ DNA in the two examples.

After restriction digestion and exonuclease treatment, minicircles are purified by phenol/chloroform extraction and ethanol precipitation. Aliquots of purified DNA are quantified by standard OD readings and total yields calculated accordingly. We have added a note in line 218. The purified seamless vector was shown in lane no. 5 & 6 of Figure 3 (B) and lane no. 6 of Figure 4 (B).

9. Line 203: From lane 4 and lane 5 in Fig. 4, if the ‘majority’ of the DNA recombined, there should be only two bands. However, there are four bands with similar luminance. Majority seems to be improper. If my understanding is right, that would indicate the recombination mediated by IntC3 in cells is not thorough as expected. Could the author give some reasons or discuss it in the discussion section?

The combined intensity of the two expected recombination products (band 10.3kb for linear seamless vector and 3.1kb for linear bacterial backbone) is much higher than the combined intensities of unrecombined plasmid (bands 7.8kb and 5.6kb from unrecombined plasmid). Hence, we stated that the majority of the substrate plasmid had undergone recombination.
The figure legend for Figure 4 has been amended in line 310-319.

**Reviewer #2: Comments:**

1. Line 116: The authors mentioned “degrees” instead of its symbol as used elsewhere in the manuscript.

We have followed this suggestion and incorporated the change in the amended manuscript in line 115.

2. Line 162, 179, 181, 204: “digestion” instead of “digest”.

We have followed this suggestion and incorporated the change in the amended manuscript in Line 162, 179, 181, 204

3. Line 191: Add a space between “13.4” and “kb”.

We have followed this suggestion and incorporated the change in the amended manuscript in line 191.

4. Line 198: “was analyzed” instead of “analyzed”.

We have followed this suggestion and incorporated the change in the amended manuscript in line 198.

5. Units at some places have a space between number and units and some places do not have it. Please maintain uniformity throughout the manuscript.

We have followed this suggestion and incorporated the change in the amended manuscript.

6. At some places, the authors have written “lambda integrase” and at some places with the symbol lambda. Please maintain uniformity throughout the manuscript. Check for similar errors and edit it.

We have followed this suggestion and incorporated the change in the amended manuscript.

**References:**

K F Jensen. The Escherichia coli K-12 "Wild Types" W3110 and MG1655 Have an rph Frameshift Mutation That Leads to Pyrimidine Starvation Due to low pyrE Expression Levels. J Bacteriol. 1993; 175(11): 3401–3407. doi: 10.1128/jb.175.11.3401-3407. PubMed PMID: 8501045.

Liu D and Reeves PR. Escherichia coli K12 regains its O antigen. Microbiol. 1994; 140: 49-57. doi.org/10.1099/13500872-140-1-49. PubMed PMID: 7512872.

Makhija H, Roy S, Hoon S, Ghadessy FJ, Wong D, Jaiswal R, et al. A novel lambda integrase-mediated seamless vector transgenesis platform for therapeutic protein expression. Nucleic Acids Res. 2018;46(16):e99. Epub 2018/06/13. doi: 10.1093/nar/gky500. PubMed PMID: 29893931; PubMed Central PMCID: PMCPMC6144826

R P Lawther, D H Calhoun, J Gray, C W Adams, C A Hauser, G W Hatfield. DNA Sequence Fine-Structure Analysis of ilvG (IlvG+) Mutations of Escherichia coli K-12. J Bacteriol. 1982 Jan; 149(1): 294–298. doi: 10.1128/jb.149.1.294-298.1982. PubMed PMID: 7033211.

Vijaya Chandra SH, Makhija H, Peter S, Myint Wai CM, Li J, Zhu J, et al. Conservative site-specific and single-copy transgenesis in human LINE-1 elements. Nucleic Acids Res. 2016;44(6):e55. Epub 2015/12/18. doi: 10.1093/nar/gkv1345. PubMed PMID: 26673710; PubMed Central PMCID: PMCPMC4824084.
